# Physical and Functional Properties of Powders Obtained during Spray Drying of *Cyani flos* Extracts

**DOI:** 10.3390/molecules29143400

**Published:** 2024-07-19

**Authors:** Katarzyna Lisiecka, Dariusz Dziki, Monika Karaś

**Affiliations:** 1 Department of Biochemistry and Food Chemistry, University of Life Sciences in Lublin, Skromna St. 8, 20-704 Lublin, Poland; monika.karas@up.lublin.pl; 2 Department of Thermal Technology and Food Process Engineering, University of Life Sciences in Lublin, Głęboka St. 31, 20-612 Lublin, Poland

**Keywords:** inulin, pectin, edible flower, *Cyani flos*, microstructure, color, phenolic compounds

## Abstract

Edible flowers are a potential source of bioactive ingredients and are also an area of scientific research. Particularly noteworthy are *Cyani flos*, which have a wide range of uses in herbal medicine. The below study aimed to investigate the influence of selected soluble fiber fractions on the selected properties of physical and biochemical powders obtained during spray drying a water extract of *Cyani flos*. The drying efficiency for the obtained powders was over 60%. The obtained powders were characterized by low moisture content (≤4.99%) and water activity (≤0.22). The increase in the addition of pectin by the amount of 2–8% in the wall material resulted in a decrease in hygroscopicity, water solubility, and protection of flavonoids and anthocyanins both before and after digestion in the tested powders in comparison to the sample with only inulin as a carrier. Additionally, it was noted that all samples were characterized by high bioaccessibility when determining antioxidant properties and xanthine oxidase inhibition.

## 1. Introduction

*Cyani flos* (bachelor’s button) is an annual plant that blooms between June and August. The flower has characteristic blue petals, which owe their color to a pigment called protocyanin. The plant can often be seen in corn fields (that is why it is also called cornflower) [1,2]. It is classified as herbal medicine, with bioactive substances identified in the plant including, among others, flavonoid aglycons (quercetin, kaempferol, isorhamnetin, apigenin, luteolin, and hispidulin), flavonoid glucosides (quercetin, kaempferol, isorhamnetin, apigenin, luteolin, and hispidulin) and hydroxycinnamic acids [3]. Due to the content of bioactive compounds, the plant has health-promoting effects, including inflammatory, antimicrobial, antipruritic, antitussive, astringent and many other biological activities [1]. In recent years, there has been an increase in interest in natural products due to increased consumer awareness. Therefore, edible flowers can be an additional source of health-promoting ingredients, not only in the form of infusion [1,4,5].

Spray drying is an economical method of encapsulating bioactive substances; however, to avoid unfavorable conditions during dehydration and storage losses, a carrier must be introduced into the system [6]. Popular wall materials in the encapsulation process are carbohydrate-based biopolymers, i.e., soluble fiber fractions. The most noteworthy ones include inulin and pectin. Despite its hydrophilic nature, inulin is suitable for protecting hydrophobic substances [7], i.e., anthocyanins [8]. Inulin has been used in the literature as a probiotic, but also as a sugar and fat substitute and as a texture modifier in broadly understood functional foods [9]. Nevertheless, it should be mentioned that inulin microcapsules are sensitive to environmental conditions, which is why they are often combined with other carriers [7]. Lisiecka et al. [4] noticed the benefits of mixing inulin and pectin; it reduces the tendency of powders to agglomerate and has a protective effect on flavonoids and anthocyanins in general. It is worth mentioning that pectins are known for their biological effects, including the ability to lower cholesterol, glucose and insulin levels [10]. However, regardless of the carriers chosen, the physical properties of the powder should be examined and the impact of the wall material on the active substances, protection ability, and potential bioaccessibility should be examined [4]. This is especially since the production of functional nutritional preparations with bioactivity components involves many problems, including loss of biological activity, sensitivity to environmental conditions and unsatisfactory properties of the final product [6].

This work aimed to investigate the influence of selected fiber fractions on the physical and biochemical properties of powders obtained from *Cyani flos* infusions during spray drying.

## 2. Results and Discussion

It is assumed that the spray drying process has been carried out correctly if the recovery iss 50% or more [11], but this value should be treated only as a reference value because the small diameters of drying chambers in a laboratory scale favor the sticking of partially moist particles [12]. The introduction of pectin into the composition of the carrier resulted in a reduction in the process yield (PY) of the process in comparison to the sample with only inulin in the wall material. Significant differences in the amount of recovered powder were observed only when pectin replaced inulin at an amount of 6% (Table 1). In the case of spray drying, powder recovery is influenced by the material carrier used [13]. The spray drying temperature is higher than the glass transition temperature [14], which, in the case of inulin, with a degree of polarization (DP) less than or equal to 10, the glass transition temperature (Tg) is approximately 79.68 °C, and in the case of DP > 23, it is 118.85 °C [15], while in the case of pectin, regardless of the degree of esterification, Tg is 35 °C [16]. Therefore, a transition to a rubbery viscoelastic state occurs, which is observed as drying material sticking to the chamber [17].

Additionally, it was noticed that PY was positively correlated with WA (r = 0.72, *p* = 0.174), H (r = 0.87, *p* = 0.056) and S (r = 0.94, *p* = 0.016) presented by the powders, but only in the last case was the relationship linearly significant, which generally indicates that the relationship of these parameters results from differences in the composition of the wall, which is associated with the introduction of pectin in favor of inulin.

### 2.1. Physical Properties of Powders

The basic physical properties of powders based on a *Cyani flos* extract are presented in Table 1. The moisture content (MC) of powders was between 4.45% and 4.99% (Table 1). Authors dealing with powders assume that low water content is important for their stability. It is assumed that the moisture content of powders should not exceed 5% and the water activity should not exceed 0.6 [17]. Water activity (WA) is a measure of the availability of water that can potentially enter into a biochemical reaction [18]. The water activity of powders was from 0.16 to 0.22 (Table 1). High hygroscopicity translates into higher water absorption and causes the stickiness of powders. Therefore, low hygroscopicity of powders is desired [19]. In the case of solubility (S), its value is considered in terms of further use and intended use of the powder. If we want the powder to be used for the production of various food products, high solubility will be desirable [14]. In the case of hygroscopicity (H) and solubility, the addition of pectin to the carrier decreased the hygroscopicity and solubility of the powders. However, the replacement of inulin by pectin at amounts of 6% and 8% had a significant impact on the tested parameters, respectively (Table 1). Also, it was observed that a positive correlation was between H and S (r = 0.83, *p* = 0.082), and a negative correlation between S and the 10th percentile of size particles of powders (r = −0.79, *p* = 0.112); however, in both cases, it was not linearly significant.

### 2.2. Color Profile of Powders

Powders with the addition of edible flower infusion, which included pectin as a carrier component, were significantly darker and redder than the control sample, which contained only inulin as a carrier component (Table 2). Additionally, a strong significant correlation was observed between the L* coordinate and the total flavonoid content (r = −0.89, *p* = −0.043), as well as the a* coordinate and the anthocyanin content (r = 0.90, *p* = 0.035), and additionally a strong correlation was observed between L* and a* (r = −0.99, *p* = 0.002). Cornflower flowers contain a blue protocyanin pigment as a metalloanthocyanin complex formed by cyanidin−3-O-(6-O-succinylglucoside)-5-O-glucoside,apigenin-7-O-glucuronide-4′-O-(6-O-malonylglucoside), Fe^3+^ and Mg^2+^ in the stoichiometric order 6:6:1:1. The bonds in this complex are so weak that the pigment ceases to be stable after dilution [2]. Additionally, in our case, we prepared an infusion using distilled water with a slightly acidic pH, which explains the redness of the powders when anthocyanins are present in the pigments. However, the increasing redness of the samples is probably related to the increasing amount of pectin, which has a protective effect in the case of anthocyanins [4,20]. Despite color changes, the ∆*E* index was so low that the difference between powders could only be distinguished by observers in cases C4 and C5 (∆*E* = 1.5–5) [14].

### 2.3. Particle Structure of Powders with Inulin and Pectin as a Wall Core

**Figure 1:**
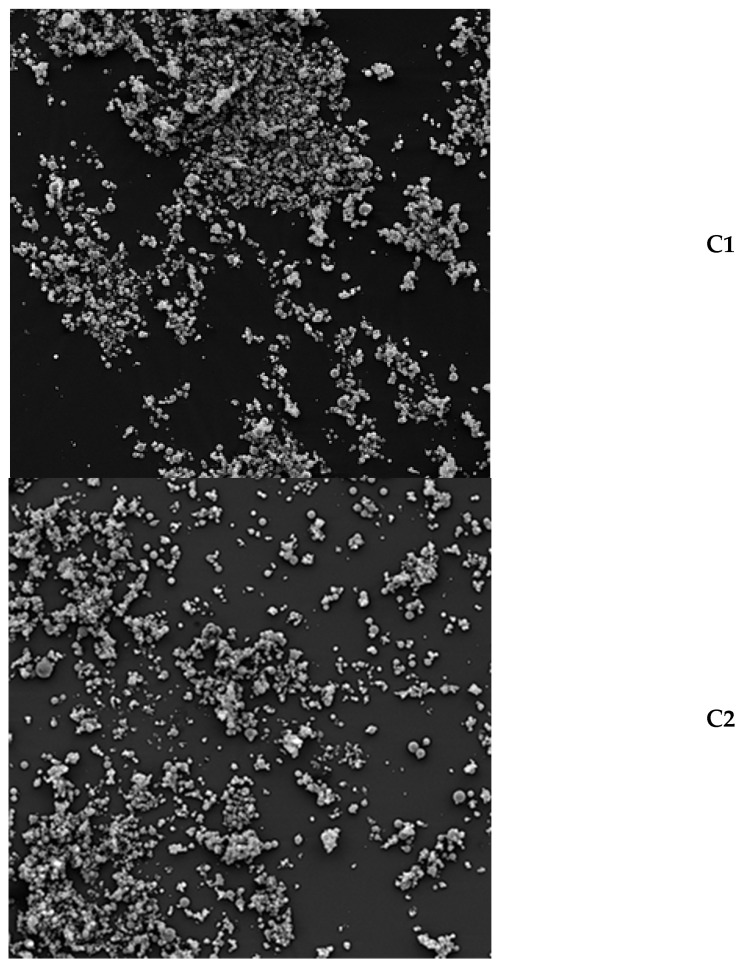

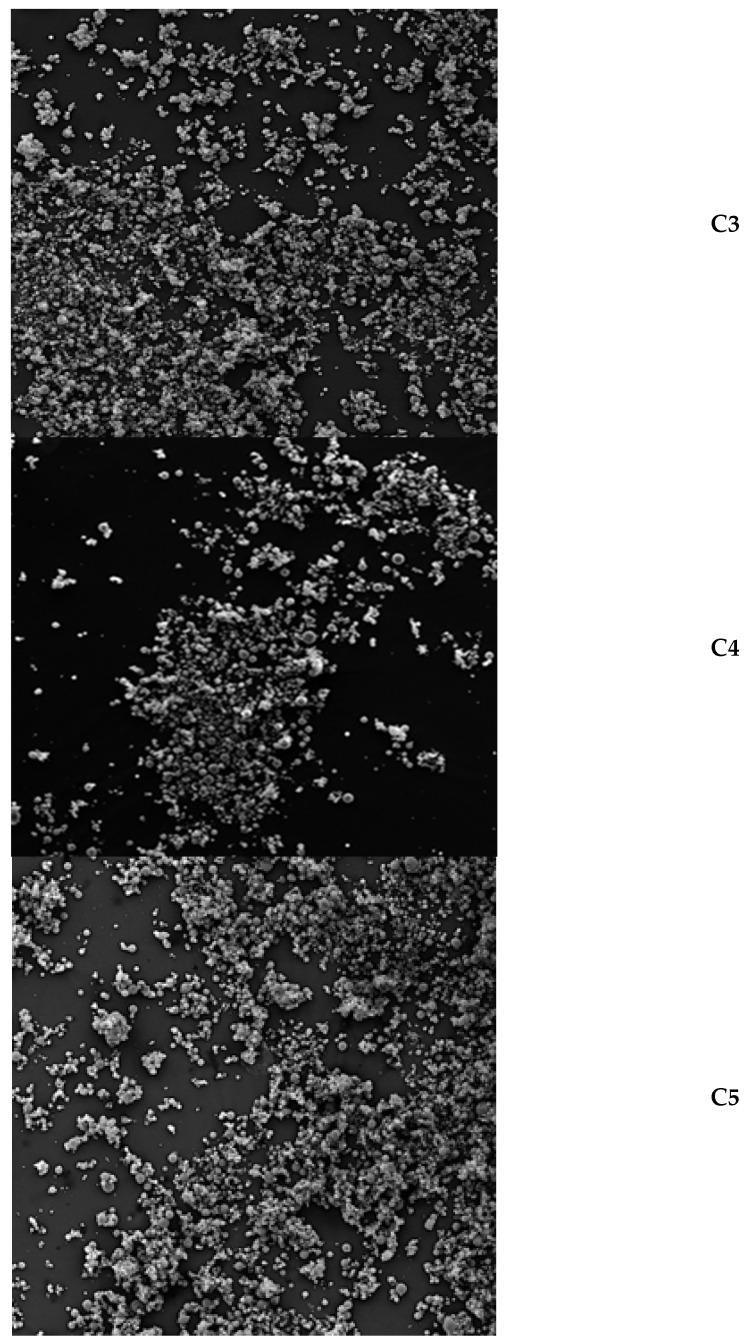
The magnification (500×) of powders from *Cyani flos* made with SEM: C1—control sample with the addition of 0% pectin (100% inulin), C2—sample with the addition of 2% pectin (98% inulin), C3—sample with the addition of 4% pectin (96% inulin), C4—sample with the addition of 6% pectin (94% inulin), C5—sample with the addition of 8% pectin (92% inulin).

**Figure 2:**
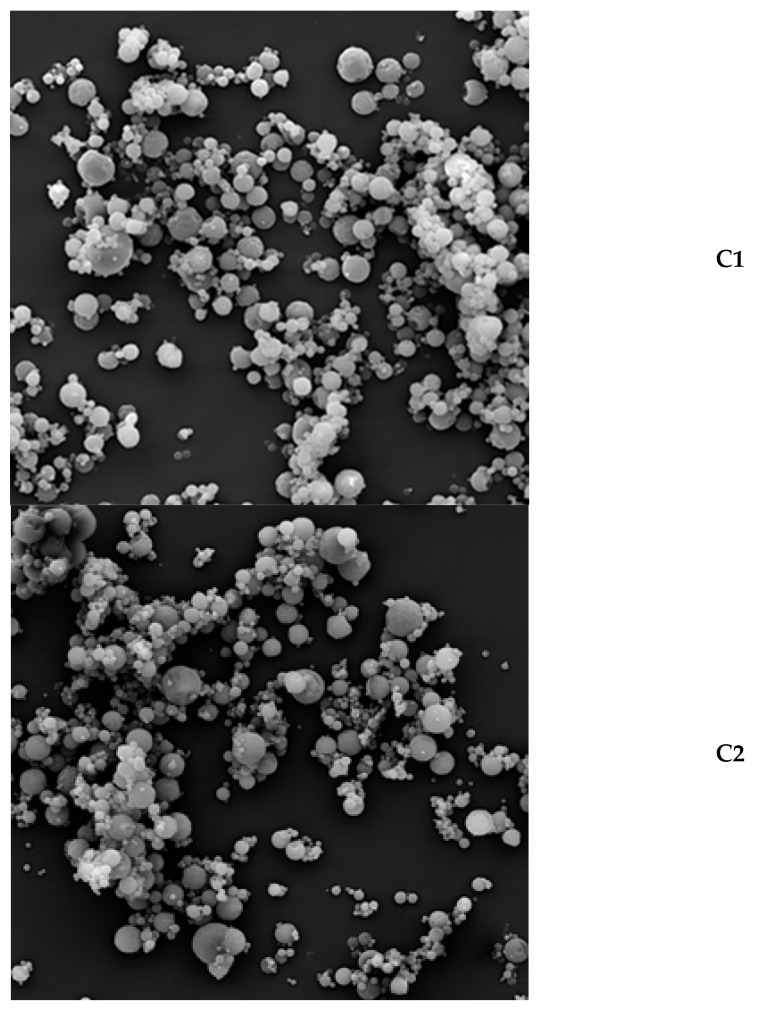

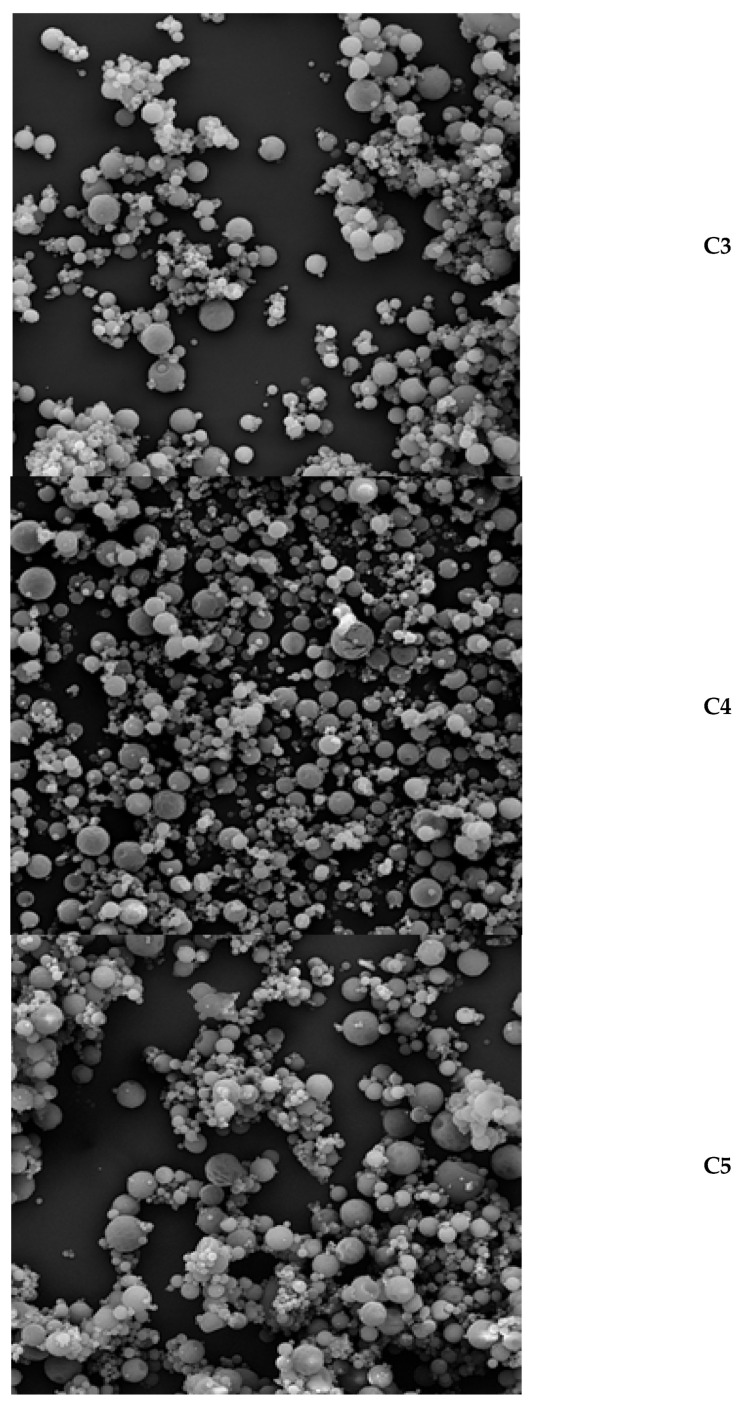
The magnification (2000×) of powders from *Cyani flos* made with SEM: C1—control sample with the addition of 0% pectin (100% inulin), C2—sample with the addition of 2% pectin (98% inulin), C3—sample with the addition of 4% pectin (96% inulin), C4—sample with the addition of 6% pectin (94% inulin), C5—sample with the addition of 8% pectin (92% inulin).

Magnification (500×) showed that the tested powders which were dried at 140 °C tended to agglomerate (Figure 1). At ×2000 magnification, we can observe that the vast majority of particles had a spherical and smooth shape with a few cracks and irregularities (Figure 2). Researchers drying apple juice concentrate with the addition of maltodextrin and pectin also noticed globular particles with smooth surfaces and many dents. They attributed the problem in creating a uniform spherical structure to pectin, which increases viscosity and gel formation ability that prevents the formation of particles with uniform walls [21].

Scientists who spray-dried pineapple extract using inulin at inlet temperatures of 150 °C and 190 °C noticed a smoother surface and a higher degree of agglomeration when the inulin was dried with the extract than alone [22]. In the literature, you can find information that the morphology of inulin during spray drying is influenced primarily by the temperature of the feed solution and the inlet temperature. The increase in these parameters increases the amorphous structure in the dried product. It was noticed that a drying temperature below 200 °C does not cause any damage to the particle [23]. Quintriqueo-Cid et al. [24] suggest that the morphology of microparticles would be attributed to inulin conformation. If we observe spherical shapes and a smooth surface, we are dealing with its amorphous form, while when irregular surfaces and numerous roughness levels occur, semicrystalline microparticles are observed.

### 2.4. Particle Size Distribution of Powders

According to the data in Table 3, the 10th percentile value for the tested powders ranged from 0.52 to 0.59 µm. In this case, the 50th and 90th percentile values were 3.68 to 3.92 µm and 9.71 to 12.97 µm, respectively. However, these were not statistically significant differences, generally. Also, for the span index, no significant differences were observed between the tested powders, which indicates their slight differentiation. Authors dealing with the subject of spray drying report that the particle size of the final powder depends on the structure of the polymer; the shorter the chain, the smaller the particles [25]. Additionally, others have noticed that the particle diameter is also influenced by variable drying parameters, the atomization method used, the carrier used (type and concentration) or the viscosity of the dried material [14]. Researchers observing the span index in the range of 1.75–2.47 define its value as low, which indicates a homogeneous distribution of powders after spray drying [26].

### 2.5. Bioactive Compounds and Antioxidant and Anti-Inflammatory Properties of Powders

The bioactive compounds of powders with *Cyani flos* extracts with antioxidant and anti-inflammatory properties are presented in Table 4. The total content of flavonoids and anthocyanins before and after digestion was significantly higher in the samples in which a pectin and inulin mix was used as a carrier than in the samples in which only inulin was applied. Additionally, a strong positive and significant correlation was observed between B-TF and B-TA and the increasing amount of pectin in the capsule core (r = 0.93, *p* = 0.020 and r = 0.90, *p* = 0.038, respectively). Authors using inulin/fructooligosaccharides/pectin-based structured systems when encapsulating bioactive ingredients from jussara pulps observed that pectin combined with inulin or fructooligosaccharides protected polyphenolic compounds better than when used alone as a carrier [27]. This is due to the formation of a branched structure in which the availability of bonds increases, which are responsible for the mechanism of interaction between polyphenolic compounds and the microstructure of carrier blends [4,26]. Sansone et al. [28], during a microstructure study, found that the combination of pectin and maltodextrin provided better protection for polyphenols than maltodextrin alone because, in such a combination, pectin was a coating agent and maltodextrin acted mainly as a substance responsible for creating the matrix. Bioaccessibility for flavonoids and anthocyanins ranged from 0.77 (C1) to 0.99 (C3), and from 0.58 (C1) to 0.85 (C5), respectively (Table 4). The decrease in the content of polyphenol compounds after digestion results from the sensitivity of these compounds to an alkaline environment [29,30]. As a result, at the intestinal stage, anthocyanin rings are split, which results in degradation and, consequently, a decrease in the anthocyanin content. Additionally, anthocyanins interact with enzymes and bile salts, resulting in the formation of insoluble complexes [31].

The ABTS radical scavenging (AB) value was higher for all samples after digestion in comparison to this same sample before digestion (Table 4). As a result, the bioaccessibility of AB ranges from 1.15 (C5) to 2.33 (C2). Álvarez-Cervantes et al. [32] claimed that an increase in the content of polyphenols and antioxidant activity after digestion may indicate effective encapsulation. In our case, we only observe an increase in antioxidant activity and a decrease in the total content of flavonoids and anthocyanins after digestion. Researchers determining the antioxidant activity of flavonoid aglycones and glycosides, similarly to our case, noticed increases in antioxidant activity in the intestinal phase, but only in the case of isoquercitrin (O-glycoside) and orientin (C-glycoside) in comparison the original form before digestion, which was due to the instability of aglycones during digestion [33]. It is worth emphasizing those flavonoid aglycons (quercetin, kaempferol, isorhamnetin, apigenin, luteolin, hispidulin), and their glycosides were identified in *Centaurea cyanus* L. [3]. Generally, polyphenols are dependent on the alkaline pH of the intestine; a significant part of the compound may be changed into other unknown and/or undetected structural forms with different chemical properties and, consequently, different bioaccessibility, bioavailability and biological activity [34]. Due to the dependence on pH, an increase in antioxidant properties can be observed, because polyphenols after deprotonation donate electrons more easily and are more stable at alkaline pH, which leads to polymerization. Consequently, we can talk about new oxidizable OH moieties in their polymeric products [35]. In the case of F, only for the sample containing inulin in the carrier composition was an increase in the value after digestion observed. Therefore, the C1 sample was characterized by the highest bioaccessibility. Lisiecka et al. [4], who tested microcapsules with the addition of mallow extract, also observed the highest bioaccessibility for the fractions responsible for ferric-reducing antioxidant power for samples containing only inulin. In the case of encapsulation of polyphenols extracted from jabuticaba (*Myrciaria jabuticaba* (*Vell.*) Berg) peel, it was noticed that when using a carrier in the form of inulin and pectin, a higher ferric-reducing antioxidant power was observed than when using pectin alone [27].

The search for potential xanthine oxidase inhibitors is important because overactivity of the enzyme may lead to oxidative stress and related diseases [4]. It is also crucial to look for lipoxygenase inhibitors because the enzyme is associated with inflammatory joint diseases, asthma, psoriasis, intestinal inflammation and neurodegenerative disorders such as Alzheimer’s [36]. Samples containing pectin had a stronger ability to inhibit the activity of xanthine oxidase (IXO) and lipoxygenase (ILOX) before digestion and after digestion (only for IXO). The ability to inhibit lipoxygenase after digestion between powders was varied, but differences in inhibitory ability were statistically insignificant. In the case of A-IXO, a significant and positive correlation was observed between TF (r = 0.93, *p* = 0.024 and r = 0.95, *p*= 0.013) and TA (r = 0.97, *p* = 0.006 and r = 0.96, *p* = 0.009) before and after digestion, respectively; it can be assumed that flavonoids and anthocyanins present in pansy flowers are potential xanthine oxidase inhibitors. The methanol extract of Roselle flower (*Hibiscus sabdariffa* L.) was examined, and it was observed that fraction 2, potentially referred to as anthocyanins, also exhibits xanthine oxidase inhibitor potential [37]. Due to their structure and free hydroxyl groups, flavonoids can scavenge free radicals as well as participating in metal chelation and lipoxygenase inhibition [38]; however, in our case, there was no significant association between B/A-ILOX and the content of flavonoids or anthocyanins.. The bioaccessibility for IXO was between 1.27 (C4) and 1.65 (C1); in the case of ILOX, it was twice as high for C1 than the other tested samples. Scientists who digested bread with the addition of Saskatoon berry observed an increase in the ability to inhibit lipoxygenase after digestion (bioaccessibility > 1), indicating that such products may translate into the prevention of the formation of reactive oxygen species [39].

## 3. Materials and Methods

*Cyani flos* was supplied by ECOHERBA (Hajnówka, Poland). Chicory inulin DP 10 (Agnex, Białystok, Poland) and amidated pectin DE 31 (C&G Sp. z o.o., Jasło, Poland) were used as carrier agents.

### 3.1. The Process of Obtaining Powder from Cyani flos

In the first step, an infusion of the edible flower was made at a temperature of 95 °C in the ratio of 1:20 *w*/*v*. The mixture was left to cool at room temperature, strained from the solids and centrifuged using an MPW-260R centrifuge (MPW, Warsaw, Poland). The supernatant was poured off and freeze-dried at −40 °C. The water extract of final lyophilisate was characterized by the following values: the total flavonoid content (TFC) was 81.28 mg quercetin/g solids, the total anthocyanin content (TAC) was 2.95 mg cyanidin 3-O-glucoside/g solids, ABTS radical scavenging was 98.24 mg Trolox/g solids, ferric-reducing antioxidant power was 55.86 mg Trolox/g solid, inhibition of xanthine oxidase was 1.53 IU/g solids, and inhibition of lipoxygenase was 3.77 kIU/g solids. The final lyophilisate with inulin and pectin was used to prepare to feed solution. The water solution of CF (1% concentration) and carrier agent (12.5% concentration) was mixed in the ratio 1:5 (*v*/*v*). The amount of CF was constant in all powders; however, the content of inulin and pectin was consistent with the entry in Table 5.

The blends were homogenized for 10 sec before the spray drying. An MBL-03 hand blender (MPW, Milanówek, Poland) was used. The input temperature was 140 °C, while the output temperature was 50 °C. The solution feed rate by the peristaltic pump was 8 mL/min. The drying air and compressed air were supplied at 38 m^3^/h and 0.742 m^3^/h, respectively. The internal diameter of the nozzle spray was 0.7 mm. The process parameters were selected experimentally. A Mini Spray Dryer Büchi B-290 (Büchi Labortechnik AG, Flawil, Switzerland) was used as the main equipment in this experiment. After obtaining the powder, PY was determined as the ratio of the mass of collected powder to the amount of solid substance that was in the solution prepared for drying. The powders awaited further analysis at a temperature of −20 °C, secured by placing them in plastic bags.

### 3.2. Physical Properties of Powders

#### 3.2.1. Moisture Content (MC) and Water Activity (WA) of Powders

A Sartorius MA35 moisture analyzer (Göttingen, Germany) and LabMaster-aw (Novasina AG, Switzerland) were used to determine MC and WA, respectively. In each instance, 0.1 g of the substance was used and the analysis was conducted automatically.

#### 3.2.2. Hygroscopicity (H) of Powders

The level of H was determined by placing 1 g of sample in the climate chamber MLR-352H-PE phcbi (Etten-Leur, The Netherlands) and maintaining a temperature of 25 °C and humidity of 75.3% for 7 days [40]. After the experiment, the sample was weighed to calculate the amount of water adsorbed per 100 g of the sample.

#### 3.2.3. Solubility (S) of Powders

To summarize, 0.5 g of the sample was dissolved in 50 mL of distilled water and agitated for 30 min using a Multi Rotator RS-24 (BIOSAN, Otwock, Poland). The mixture was then centrifuged at 5000 rpm using an MPW-352R centrifuge (MPW, Warsaw, Poland). The resulting supernatants were then dried in an oven at 105 °C until a constant weight was achieved. The S of the sample was determined by calculating the percentage of the dried weight of the soluble portion to the initial weight [41].

#### 3.2.4. The Color Profile of Powders

The color profile of powders was described by L* (balance between white and black), a* (balance between red and green) and b* (balance between blue and yellow) coordinates; additionally, ∆*E* was calculated. A portable calorimeter, NH310 (EnviSense, Lublin, Poland), was used for measurement on the C-Lab scale [42].

#### 3.2.5. The Particle Size Distribution of Powders

The Malvern Mastersizer 3000, a laser particle size analyzer, was used to measure the particle size distribution (PSD) of the powders. The analysis involved laser light scattering ranging from 0.01 mm to 3.0 mm; 5 g of the sample was prepared for analysis, which was conducted automatically using laser diffraction and the dry dispersion method. The results of the particle size distribution survey were presented as d10, d50 (median diameter), and d90, representing the 10th, 50th, and 90th percentile of the total volume of particles, assumed to be spherical in this study [43].

#### 3.2.6. Microstructure of Powders

The scanning electron microscope VEGA LMU (TESCAN, Brno, Czech Republic) working at an acceleration voltage of 10 kV was used as tool to research the microstructure of the powders. Beforehand, powders were frozen in liquid nitrogen and then lyophilized. A single sample of powder was placed on a carbon disk using special silver tape and covered with gold in a vacuum sublimation K-550× (Emitech, Ashford, UK). Pictures were taken at ×500 and ×2000 magnification [4].

### 3.3. Biochemical Determination of Properties of Powders

#### 3.3.1. Extraction of Powders

Aqueous extracts were made using 5 mL of distilled water and weighing 0.4 µg of the sample each time. A Multi RS-60 rotator (Biosan, Otwock, Poland) was used to shake the samples; this step was performed for 30 min. Then, the MPW-352R centrifuge (MPW, Warsaw, Poland) was used for centrifugation with the following parameters: 4 °C for 10 min at 5000 rpm. Extractions were performed twice and the obtained supernatant was kept at −20 °C.

#### 3.3.2. In Vitro Digestion of Powders

The simulated digestion was performed based on the methodology proposed by Minekus et al. [44] and Lisiecka et al. [4]. Vortexing, centrifugation and holding of the supernatant were performed as described in Section 3.3.1.

#### 3.3.3. The Content of Bioactive Substances of Powders

The TF [45] assay steps consisted of mixing 20 µL, 180 µL and 10 µL of 5% extract, water and sodium nitrate, respectively. After 5 min, the mixture was supplemented with 10 µL of 10% aluminum chloride; then, after 6 min, 40 µL of 1M sodium hydroxide was added. Absorbance was measured at 510 nm after 20 min. In the case of TA, the absorbance measurement was carried out at two wavelengths, 520 nm and 700 nm, because two solutions were made; the first one consisted of an extract mixed with 80 µL of 0.025 M chloride potassium buffer at pH 1, and the next one consisted of a solution containing the extract and 80 µL of 0.04 M sodium acetate buffer at pH 4.5. TA was calculated according to the formula proposed by Kopjar et al. [46].

#### 3.3.4. Antioxidant Activity against the ABTS Radical

The ABTS radical scavenging activity was measured by following the method described by Re et al. [47]. A volume of 10 µL of either the extract or solvent (used as a control) was combined with 250 µL of 7mM ABTS solution. The absorbance of the radical was set at 0.700. After incubation for 10 min, the measurement for each sample was performed at a wavelength of 732 nm.

#### 3.3.5. Ferric-Reducing Antioxidant Power (F) Assay of Powders

The analysis was performed at a wavelength of 732 nm. The analysis consisted of the following stages. Before the first incubation, which lasted 10 min at 50 °C, a three-component mixture was prepared. Each substance was added in an amount of 50 µL, including 200 mM (pH 6.6) phosphate buffer, 1% potassium ferrocyanide and the tested extract. After incubation, 50 µL of 10% TCA was added to the mixture. Half of the blend was then taken from the mixture and placed on a 96-well plate, adding 100 µL of distilled water and 20 µL of 0.1% FeCl_3_. The absorbances were then measured [48].

#### 3.3.6. Inhibition of Xanthine Oxidase (IXO)

XO activity was measured according to Habza-Kowalska et al. [49] with some modifications. First, a reaction mixture was created consisting of 20 µL of the XO enzyme, 140 µL of phosphate buffer and 20 µL of the tested extract, which acted as an inhibitor. The entire mixture was heated at 30 °C for 10 min. After incubation, 120 µL of substrate was added and incubated under the same conditions as before, and absorbance was measured at 295 nm.

#### 3.3.7. Inhibition of Lipoxygenase (ILOX)

In the first stage, an enzyme–phosphorus buffer–inhibitor mixture was created by mixing 10 µL of LOX, 240 µL of buffer and 10 µL of the extract being tested. Then, incubation was carried out for 5 min at 30 °C, which was repeated after adding 40 µL of 2.5 mmol/L linoleic acid as a substrate. The absorbance measurement was made at 234 nm [50].

#### 3.3.8. Relative Bioaccessibility

To determine the in vitro bioacceptability, the RBF (relative bioaccessibility factor) and RAF (relative antioxidant/anti-inflammatory efficiency factor) were calculated by comparing the TF, TA, AB, F, IXO, and ILOX before and after digestion [20].

### 3.4. Statistical Analysis

Statistical analysis was performed using Statistica 10.0 software (StatSoft, Inc., Tulsa, OK, USA). The post hoc Tukey test was used to compare means, using the one-way analysis of variance (ANOVA), and correlations between dependent and non-dependent variables were examined in both cases by α = 0.05.

## 4. Conclusions

The presented research results showed that it is possible to obtain powders based on an aqueous extract of *Cyani flos* edible flowers and a mixture of carrier substances, inulin and pectin. It has been proven that replacing inulin with pectin in an amount of up to 8% by mass does not negatively affect powder recovery. Additionally, powders were characterized by low moisture content and water activity. The addition of pectin had a positive effect on the properties of powders, reducing their hygroscopicity, but at the same time reducing their solubility; the powders also did not differ in color. The resulting powders tend to agglomerate regardless of the composition of the carrier. Samples in which pectin was added in amounts ranging from 2 to 8% had a positive effect on the content of bioactive components before and after digestion. It was also noticed that the obtained powders showed an increase in the ability to scavenge free radicals against ABTS and inhibit xanthine oxidation after digestion.

## Figures and Tables

**Table 1:** Selected physical properties characterizing powders based on *Cyani flos* extract with the addition of soluble fiber fraction as a carrier.

TS	PY (%)	MC (%)	WA (-)	H (g/100 g)	S (%)
C1	69.93 ± 1.12 ^b^	4.72 ± 0.06 ^b^	0.22 ± 0.00 ^d^	15.48 ± 0.30 ^b^	91.10 ± 0.90 ^b^
C2	68.63 ± 1.47 ^ab^	4.99 ± 0.03 ^c^	0.21 ± 0.00 ^c^	14.89 ± 0.20 ^ab^	87.39 ± 0.85 ^ab^
C3	68.89 ± 0.98 ^ab^	4.45 ± 0.03 ^a^	0.22 ± 0.00 ^d^	14.59 ± 0.05 ^ab^	89.83 ± 1.83 ^b^
C4	63.14 ± 1.15 ^a^	4.72 ± 0.03 ^b^	0.16 ± 0.00 ^a^	14.07 ± 0.30 ^a^	85.16 ± 0.84 ^ab^
C5	62.03 ± 1.08 ^a^	4.97 ± 0.03 ^c^	0.20 ± 0.00 ^b^	14.20 ± 0.17 ^a^	83.18 ± 0.82 ^a^

TS—type of sample, C1—control sample with the addition of 0% pectin (100% inulin), C2—sample with the addition of 2% pectin (98% inulin), C3—sample with the addition of 4% pectin (96% inulin), C4—sample with the addition of 6% pectin (94% inulin), C5—sample with the addition of 8% pectin (92% inulin); ^a–d^—means described with the same signs in the column do not show statistical differences (α = 0.05); n = 3 ± standard deviation.

**Table 2:** Color coordinates for powders obtained from *Cyani flos* with the addition of soluble fiber fractions.

TS	L* (*-*)	a* (*-*)	b* (*-*)	∆*E* (*-*)
C1	95.24 ± 0.15 ^c^	1.38 ± 0.05 ^a^	−5.04 ± 0.11 ^ab^	-
C2	94.63 ± 0.30 ^b^	2.18 ± 0.14 ^b^	−4.71 ± 0.32 ^a^	1.01
C3	94.66 ± 0.21 ^b^	2.04 ± 0.07 ^b^	−5.20 ± 0.16 ^b^	0.88
C4	94.09 ± 0.09 ^a^	2.64 ± 0.03 ^c^	−4.79 ± 0.02 ^a^	1.71
C5	94.29 ± 0.37 ^ab^	2.62 ± 0.04 ^c^	−4.92 ± 0.15 ^ab^	1.58

TS—type of sample, L*—the balance between white and black, a*—redness, b*—blueness, ∆*E*—index describes the total color difference, C1—control sample with the addition of 0% pectin (100% inulin), C2—sample with the addition of 2% pectin (98% inulin), C3—sample with the addition of 4% pectin (96% inulin), C4—sample with the addition of 6% pectin (94% inulin), C5—sample with the addition of 8% pectin (92% inulin); ^a–c^—means described with the same signs in the column do not show statistical differences (α = 0.05); n = 5 ± standard deviation.

**Table 3:** Characterization of particle size distribution of *Cyani flos* powders.

TS	d_10_ (µm)	d_50_ (µm)	d_90_ µm (-)	Span (-)
C1	0.52 ± 0.02 ^a^	3.89 ± 0.07 ^a^	12.97 ± 2.85 ^a^	3.19 ± 0.68 ^a^
C2	0.59 + 0.02 ^b^	3.91 ± 0.08 ^a^	10.06 ± 0.57 ^a^	2.42 ± 0.10 ^a^
C3	0.54 ± 0.02 ^ab^	3.68 ± 0.06 ^a^	9.71 ± 0.49 ^a^	2.49 ± 0.09 ^a^
C4	0.58 ± 0.01 ^ab^	3.90 ± 0.06 ^a^	10.90 ± 0.45 ^a^	2.65 ± 0.08 ^a^
C5	0.58 ± 0.0.2 ^ab^	3.92 ± 0.21 ^a^	10.50 ± 1.93 ^a^	2.52 ± 0.36 ^a^

TS—type of sample, C1—control sample with the addition of 0% pectin (100% inulin), C2—sample with the addition of 2% pectin (98% inulin), C3—sample with the addition of 4% pectin (96% inulin), C4—sample with the addition of 6% pectin (94% inulin), C5—sample with the addition of 8% pectin (92% inulin); ^a–b^—means described with the same signs in the column do not show statistical differences (α= 0.05); n = 2 ± standard deviation.

**Table 4:** Bioactive compounds in powders based on an extract of *Cyani flos* and their potential antioxidant and anti-inflammatory properties.

TS		C1	C2	C3	C4	C5
B	TF	79.25 ±14.78 ^a^	103.64 ± 4.04 ^c^	135.34 ± 5.31 ^c^	142.66 ± 9.37 ^c^	143.87 ± 7.32 ^c^
	TA	4.48 ± 0.34 ^a^	10.22 ± 1.03 ^b^	9.99 ± 0.74 ^b^	10.50 ± 0.68 ^b^	14.29 ± 1.11 ^c^
	AB	2.12 ± 0.22 ^a^	2.34 ± 0.24 ^a^	2.60 ± 0.32 ^a^	4.47 ± 0.62 ^b^	5.68 ± 0.86 ^b^
	F	1.00 ± 0.07 ^a^	1.52 ± 0.10 ^b^	1.42 ± 0.12 ^b^	1.48 ± 0.12 ^b^	1.38 ± 0.02 ^b^
	IXO	0.17 ± 0.00 ^a^	0.19 ± 0.02 ^a^	0.18 ± 0.01 ^a^	0.23 ± 0.01 ^b^	0.20 ± 0.02 ^ab^
	ILOX	0.07 ± 0.03 ^a^	0.21 ± 0.03 ^b^	0.18 ± 0.01 ^b^	0.21 ± 0.05 ^b^	0.18 ± 0.03 ^b^
A	TF	60.96 ± 6.67 ^a^	87.38 ± 7.71 ^b^	134.12 ± 5.25 ^c^	136.15 ± 8.83 ^c^	132.09 ± 6.74 ^c^
	TA	2.62 ± 0.32 ^a^	7.56 ± 1.10 ^b^	6.12 ± 0.38 ^b^	8.61 ± 1.10 ^b^	12.15 ± 1.52 ^c^
	AB	4.41 ± 0.23 ^a^	5.44 ± 0.45 ^ab^	5.36 ± 0.63 ^ab^	5.47 ± 0.38 ^bc^	6.52 ± 0.29 ^c^
	F	1.15 ± 0.11 ^a^	1.51 ± 0.18 ^b^	1.06 ± 0.07 ^a^	1.26 ± 0.06 ^ab^	1.12 ± 0.19 ^a^
	IXO	0.28 ± 0.00 ^a^	0.29 ± 0.01 ^a^	0.29 ± 0.01 ^a^	0.29 ± 0.01 ^a^	0.29 ± 0.01 ^a^
	ILOX	0.19 ± 0.01 ^a^	0.19 ± 0.04 ^a^	0.18 ± 0.06 ^a^	0.21 ± 0.03 ^a^	0.18 ± 0.05 ^a^
RBF RAF	TF	0.77	0.84	0.99	0.95	0.92
	TA	0.58	0.74	0.61	0.82	0.85
	AB	2.08	2.33	2.06	1.22	1.15
	F	1.15	0.99	0.75	0.85	0.81
	IXO	1.65	1.51	1.63	1.27	1.48
	ILOX	2.59	0.90	0.92	0.86	1.19

TF—total flavonoid content (expressed as mg quercetin/100 g solids); TA—total anthocyanin content (expressed as mg cyanidin 3-O-glucoside/100 g solids), AB—ABTS radical scavenging (expressed as mg Trolox/g solids); F—ferric-reducing antioxidant power (expressed as mg Trolox/g solids); IXO—inhibition of the xanthine oxidase (expressed as IU/g solids); ILOX—inhibition of lipoxygenase (expressed as kIU/g solids); B—before digestion, A—after digestion, RBF—relative bioaccessibility factor, RAF—relative antioxidant/anti-inflammatory efficiency factor, TS—type of sample, C1—control sample with the addition of 0% pectin (100% inulin), C2—sample with the addition of 2% pectin (98% inulin), C3—sample with the addition of 4% pectin (96% inulin), C4—sample with the addition of 6% pectin (94% inulin), C5—sample with the addition of 8% pectin (92% inulin); ^a–c^—means described with the same signs in the column do not show statistical differences (α = 0.05); n = 3 ± standard deviation.

**Table 5:** Percentage of inulin and pectin as the carrier.

Sample	I [% *w*/*w*]	P [% *w*/*w*]
C1	100	0
C2	98	2
C3	96	4
C4	94	6
C5	92	8

I—inulin; P—pectin.

## Data Availability

The data presented in this study are available on request.
